# Comprehensive analysis on phenotype and genetic basis of Chinese Fanconi anemia patients: dismal outcomes call for nationwide studies

**DOI:** 10.1186/s12881-020-01057-3

**Published:** 2020-06-01

**Authors:** Daijing Nie, Jing Zhang, Fang Wang, Wei Zhang, Lili Liu, Xue Chen, Yang Zhang, Panxiang Cao, Min Xiong, Tong Wang, Ping Wu, Xiaoli Ma, Wenjun Tian, Mangju Wang, Kylan N. Chen, Hongxing Liu

**Affiliations:** 1 Division of Pathology & Laboratory Medicine, Hebei Yanda Lu Daopei Hospital, 6 Sipulan Road, Langfang, 065201 China; 2 Beijing Lu Daopei Institute of Hematology, Beijing, 100176 China; 3 Department of Hematology, Hebei Yanda Lu Daopei Hospital, Langfang, 065201 China; 4 Department of Clinical Laboratory Medicine, Shandong Provincial Hospital Affiliated to Shandong University, Jinan, 250000 China; 5 Department of Hematology, Peking University First Hospital, Beijing, 100034 China; 6 Division of Pathology & Laboratory Medicine, Beijing Lu Daopei Hospital, Beijing, 100176 China

**Keywords:** Fanconi anemia, Bone marrow failure, Aldehyde dehydrogenase, Hematopoietic stem cell transplantation

## Abstract

**Background:**

Fanconi anemia (FA) is the most common inherited bone marrow failure (BMF) syndrome with 22 related genes identified. The *ALDH2* rs671variant has been proved related to accelerate the progression of BMF in FA patients. The phenotype and genetic basis of Chinese FA patients have not been investigated yet.

**Methods:**

We analyzed the 22 FA-related genes of 63 BMF patients suspected to be FA. Clinical manifestations, morphological and cytogenetic feathers, *ALDH2* genotypes, treatment, and outcomes of the definite cases were retrospectively studied.

**Results:**

A total of 21 patients were confirmed the diagnosis of FA with the median age of BMF onset was 4-year-old. The number of patients manifested as congenital malformations and growth retardation were 20/21 and 14/21, respectively. BM dysplasia and cytogenetic abnormalities were found in 13/20 and 8/19 patients. All the patients with abnormal karyotypes also manifested as BM dysplasia or had evident blasts. Thirty-five different mutations were identified involving six genes and including twenty novel mutations. *FANCA* mutations contributed to 66.67% of cases. Eight patients harboring *ALDH2*-G/A genotype have a significantly younger age of BMF onset (*p* = 0.025). Within the 19 patients adhering to continuous follow-up, 15 patients underwent hematopoietic stem cell transplantations (HSCTs). During the 29 months of follow-up, 8/19 patients died, seven of which were HSCT-related, and one patient who did not receive HSCT died from severe infection.

**Conclusions:**

The phenotypic and genetic spectrum of Chinese FA patients is broad. Bone marrow dysplasia and cytogenetic abnormalities are prevalent and highly consistent. The overall outcome of HSCTs is disappointing. Nationwide multicenter studies are needed for the rarity and adverse outcome of this disease.

## Background

Fanconi anemia (FA) is a rare genetic disease highly heterogeneous in clinical manifestations and genetics. Clinical features primarily include congenital malformations, progressive bone marrow failure (BMF), and predisposition to hematopoietic and solid malignancies [1, 2]. The most common congenital abnormalities include skin pigmentation, café au lait spots, short stature, and hypoplastic of radii and/or thumbs [2]. The time of BMF onset is variable but usually at pre-school age, with the cumulative incidence of 90% by the age of 40 [3]. The malignancy risk in FA patients is mounting, especially the risks of myelodysplastic syndrome/acute myeloid leukemia (MDS/AML), which are several hundred folds higher than those of the general population [3–6].

Twenty-two genes have been identified related to FA (Table S1) to date. Products of the 22 genes participate in FA-BRCA pathway, which is responsible for correcting interstrand crosslinks (ICLs) and other DNA damage events induced by genotoxic agents. Endogenous aldehyde is a genotoxic agent and is detoxicated by aldehyde dehydrogenases (ALDHs) in vivo [7]. Previous studies have suggested aldehydes are highly toxic in FA deficient cells and could speed up the development of BMF and leukemia in FA deficient mice models [8–10]. The mitochondrial ALDH2 isoform is the most efficient acetaldehyde-detoxifying enzyme in humans [11]. Inactivating *ALDH2* variant (rs671 c.1510G > A/p.E504L) is highly prevalent in east Asia and can abolish ALDH2 activity by a dominant-negative effect [12]. *ALDH2*-A/A and *ALDH2*-G/A genotypes have been proved related to accelerated progression of BMF and malignant transformation in FA patients [13, 14].

Although the genetic basis, pathological mechanisms, and epidemiology of FA have been extensively studied, few researches focus on Chinese patients [15]. In the present study, we report 21 Chinese FA patients aiming to depict their genetic basis and clinical characteristics.

## Methods

### Patient enrollment

We retrospectively analyzed 63 BMF patients who were suspected to be inherited BMF in Hebei Yanda Lu Daopei Hospital from May 2012 to Dec. 2017. Detailed disease histories and examination files were retrieved from the electronic medical record system of our institute. BMF is considered with one or more lineages decreased in whole blood cell counts and reduced hematopoiesis with routine Wright-Giemsa staining bone marrow morphological analysis and Hematoxylin-Eosin staining pathological analysis. Categorization of hematopoietic cells, blast percentage, hematopoietic grade, dysplasia, and diagnosis of MDS were according to the 2008 edition of the World Health Organization Classification of Tumors of Hematopoietic and Lymphoid Tissues [16]. All patients enrolled were confirmed BMF and should meet at least two following inclusive criteria: 1) growth retardation; 2) congenital physical malformations; 3) early onset of BMF (≤ 6 years old); 4) chronic onset of BMF with a progressive course (disease course > 6 months); 5) suggestive family history (consanguinity or family history of cancer or hematological disorders); 6) positive for chromosome breakage test (Supplementary methods) (Table S2). Other inherited syndromes manifested as BMF and malformations such as dyskeratosis congenita, Diamond-Blackfan anemia, and Neurofibromatosis-Noonan syndrome diagnosed based on syndromic presentations combined with genetic tests were excluded. The follow-up duration was defined as the time from referral to the last follow-up or loss of follow-up/death.

Written informed consents were obtained from the patients or their statutory guardians and all tested family members in accordance with the Declaration of Helsinki. The study was approved by the ethics committee of the Hebei Yanda Lu Daopei hospital.

### Nucleic acid extraction

Peripheral blood (PB), bone marrow (BM), or cryopreserved DNA samples of the patients and their parents were obtained. Genomic DNA was extracted from PB/BM nucleated cells using silica gel column method.

### High throughput sequencing, variant calling, and *ALDH2* genotyping

We carried out Sanger sequencing on the entire coding exons and flank regions of *FANCA*, *FANCC*, and *FANCG* in patients suspected to be inherited BMF from Apr. 2012 to May 2016. Targeted high-throughput sequencing (THS) has been applied since May 2016, *FANCD2* and *BRCA2* were added in the panel. Whole genomic sequencing (WGS) was carried out using cryopreserved samples for the enrolled cases where the panel test could not find the pathogenic mutations, and all the 22 FA genes were analyzed.

The THS process has been described previously [17]. For the WGS, libraries were constructed with NEBNext Ultra II DNA Library Prep Kit for Illumina (New England Biolabs, US), followed by sequencing on Illumina HiSeq X Ten platform (Illumina, US) using HiSeq X Ten Reagent Kit v2.5 (Illumina, US) running on paired-end 150 bp mode.

Reads yielded by the two kinds of sequencing were all aligned to the human reference genome (hg19) with the Burrow-Wheeler Aligner (BWA) mem. Variants were called according to Genome Analysis Toolkit (GATK) best practices using bam files. Final confident variants were annotated using annovar and oncotator. Variants with minimal allele frequency (MAF) ≥ 1% in general population were filtered out according to 1000 Genomes, EXAC, and gnomAD databases. The pathogenicity of the germline missense mutations was assessed by in silico prediction algorithms, and the putative causal variants were classified according to the standards and guidelines recommended by the American College of Medical Genetics and Genomics (ACMG) [18]. Only pathogenic, likely pathogenic, or uncertain significance variants were considered causative in the present study. The process of CNV analysis based on WGS has been described elsewhere [17].

*ALDH2* genotyping was based on WGS data or Sanger sequencing with primers 5′- TGCTATGATGTGTTTGGAGCC-3′ (forward) and 5′-ATTTAGGGTCTCTGCTGGGCG-3′ (reverse).

### Validation by sanger sequencing

Polymerase chain reaction (PCR) and Sanger sequencing performed on the ABI 3500xL Genetic Analyzer (Thermo Fisher, US) were adopted to confirm all the mutations reported in this study. Single nucleotide variants (SNVs) and small insertions/deletions (InDels) were validated by PCR and Sanger sequencing using pedigree’s samples when accessible. For the validation of CNVs, the breakpoints were confirmed by Sanger sequencing using patients’ DNA, and the parental origins were verified through PCR and agarose gel electrophoresis.

## Results

### Demography and clinical characteristics

A total of 21 patients (six females and fifteen males) from non-related families were finally diagnosed as FA, including one who has already been reported (Case 8) [17]. The median referral age of this cohort was 7 years old, and the median age of BMF onset was 4 years old (range, 1–13 years old). There were 20 Han Chinese and one Uyghur Chinese, and the geographical distribution spread nationwide though over half of the patients came from the south or southwest of China. All patients were referred to our institute because of severe cytopenia except a thirty-year-old boy (Case 10) who was initially diagnosed as MDS for the myeloid dysplasia and increased myeloblasts indicated by BM morphology. Five patients had an indicative family history with two patients had family members died from anemia (Case 3, Case 15), two patients were from consanguineous families (Case 16, Case 21), and one patient was an in vitro fertilize baby whose paternal grandmother died from pancreatic cancer (Case 4) (Table 1).

**Table 1:** Clinical features

Case No.	Gender	Age of referral (years)	Age of clinical BMF onset (years)	Congenital malformations	Growth retardation	Family history
1	M	12	2	S, C, M	Yes	Negative
2	M	7	2	S, C, G	Yes	Negative
3	M	10	5	S, G	Yes	One sibling manifested as polydactyly and died from anemia
4	M	11	5	C, G, M,	Yes	One sibling manifested as polydactyly and died from anemia IVF and paternal grandmother died from pancreatic cancer
5	F	11	10	C, H	Yes	IVF and paternal grandmother died from pancreatic cancer Negative
6	M	7	5	S, M	No	Negative
7	M	17	10	S, M	No	Negative
8	M	7	7	S, C, M, H	No	Negative
9	M	5	4	S, M	No	Negative
10	M	13	13	E	Yes	Negative
11	M	9	6	S	Yes	Negative
12	F	7	7	M	Yes	Negative
13	M	7	1	S, M	Yes	Negative
14	M	5	2	S	No	Negative
15	M	14	5	C, G	Yes	Two family members died from anemia
16^a^	M	6	4	S, C, M	Yes	2nd degree consanguinity
17	M	4	3	None	No	Negative
18	F	9	4	S, M	No	Negative
19	F	7	4	S, G, N	Yes	Negative
20	F	6	3	S, C, G	Yes	Negative
21	F	6	4	S, M, H, E, G	Yes	2nd degree consanguinity

Skin and annex abnormalities include skin pigmentation, café au lait spots, excess hair; craniofacial anomalies include microcephalus, ptosis, hypertelorism, hypotelorism, flat nose bridge; malformations in musculoskeletal system include polydactyly, deformity of thumbs, absence of thumbs, hypoplasia of thenar eminence, and scoliosis; genitourinary system malformations include kidney malformation, hydronephrosis, indirect inguinal hernia, cryptorchidism, ovary absence, and uterine malformation/absence; cardiovascular system defects include patent ductus arteriosus and ventricular septal defect; nervous system abnormalities include encephalatrophy and moyamoya disease; endocrine system defects include hypothyroidism, primary adrenocortical insufficiency, and obesity

*F* female, *M* male, *S* skin and annex, *C* craniofacial anomalies, *M* musculoskeletal system, *G* genitourinary system, *H* cardiovascular system, *E* endocrine system, *N* nervous system, *IVF* in vitro fertilized

^a^Case 16 is of Chinese Uyghur ancestry

Fourteen (66.67%) patients were growth-retarded, and 20 (95.24%) patients manifested as congenital malformations. Congenital abnormalities in our cohort included skin pigmentation (13/21), café au lait spots (5/21), spin and limbs deformation (11/21), craniofacial malformations (8/21), genitourinary system malformations (7/21), cardiovascular system defects (2/21), nervous system diseases (2/21), and endocrine system defects (2/21) (Table 1).

Thoroughly evaluation of the hematologic phenotype is crucial to FA patients since BM dysplasia or pathological cytogenetics relate to disease progression and adverse hematopoietic stem cell transplantations (HSCT) outcomes [5, 14]. Twenty patients’ morphologic test results and nineteen patients’ cytogenetics test results before pre-HSCT conditioning regimen and/or chemotherapy were available. BM dysplasia was found in 13/20 (65%) patients, including one AML with the myeloblast count of 41% (Case 5) and one myelodysplasia with the blast count of 6% (Case 10). Karyotypes were described according to the International System for Human Cytogenetic Nomenclature 2013 [15]; at least 20 metaphases were analyzed for each assay. Cytogenetic abnormalities were found in 8/19 (42.11%) patients with clonality found in five patients, and half of the abnormal karyotypes involved chromosome 7 (− 7, 7q-, or der(7)t(1;7)) (Table 2). The cytogenetic result of Case 5 who was diagnosed as AML was 46, XX, der(7)t(1;7)(q21;q36) [19], which was confirmed to be non-constitutional by matched peripheral blood, and the karyotype of patient Case 10 was highly complex (Table 2). All the patients with abnormal karyotypes also manifested as dysplasia on bone marrow smear or had evident blasts, suggesting the initiation of clonal evolution in hematopoietic system.

**Table 2:** Bone marrow morphology, karyotype, chromosome breakage tests, and *ALDH2* genotypes

Case No.	BM morphology	BM karyotype	Chromosome breakage test	*ALDH2* genotype
1	Dysplasia	NA	Positive	G/A
2	Dysplasia	47,XY,+ 15[1]/46,XY[20]	Positive	G/A
3	Hypoplasia	normal	Positive	G/A
4	NA	NA	Positive	G/G
5^a^	AML	46,XX,der(7)t(1;7)(q21;q36)[20]	Positive	G/G
6	Hypoplasia	Normal	Positive	G/A
7	Dysplasia	46,XY,-7,+ 21[5]/46,XY[16]	Positive	G/G
8	Hypoplasia	Normal	Positive	G/G
9	Hypoplasia	Normal	Positive	G/A
10^b^	MDS	Complex	Positive	G/G
11	Hypoplasia	Normal	Positive	G/A
12	Dysplasia	Normal	Positive	G/G
13	Dysplasia	Normal	Positive	G/G
14	Dysplasia	Normal	Positive	G/A
15	Dysplasia	46,XY,del(7)(p13)[13]/46,XY[7]	Positive	G/A
16	Hypoplasia	Normal	Positive	G/A
17	Dysplasia	Normal	Positive	G/A
18	Dysplasia	46,XX,t(1;5)(p36.1;q13)[1]/46,XX[19]	Positive	G/G
19	Dysplasia	46,XX,del(14)(q24)[1]/46,XX[20]	Positive	G/G
20	Dysplasia	46,XX,del(7)(q22)[8]/46,XX,del(5)(p11)/46,XX[19]	Positive	G/A
21	Hypoplasia	Normal	Positive	G/A

Chromosome breakage tests were induced by mitomycin C

*NA* not available, *AML* acute myeloid leukemia, *MDS* myelodysplastic syndrome

^a^Case 5 is diagnosed as acute myeloid leukemia. The myeloblasts count 41% of the nucleated cells according to morphologic test of bone marrow smears

^b^Case 10 is diagnosed as myelodysplastic syndrome. His bone marrow morphology shows dysplasia was observed in his granulocytic lineage and megakaryocytic lineage with the myeloblasts count 6% of the nucleated cells. The result of his karyotype is:46,XY,dup(1)(q21q23),add(2)(p11.2),add(3)(q27),der(5)t(1;5)(q21;q35),add(20)(p12)[17]/45,XY,der(1)(?::1q42- > 1q21::1p36.3- > 1q32::1q21- > 1q44::?),add(2)(p11.2),add(3)(q27),add(4)(p16),der(5)t(1;5)(q21;q35),-18,add(20)(p12),ace [2]/46,XY [1]

### Characteristics of mutations

**Fig. 1:**
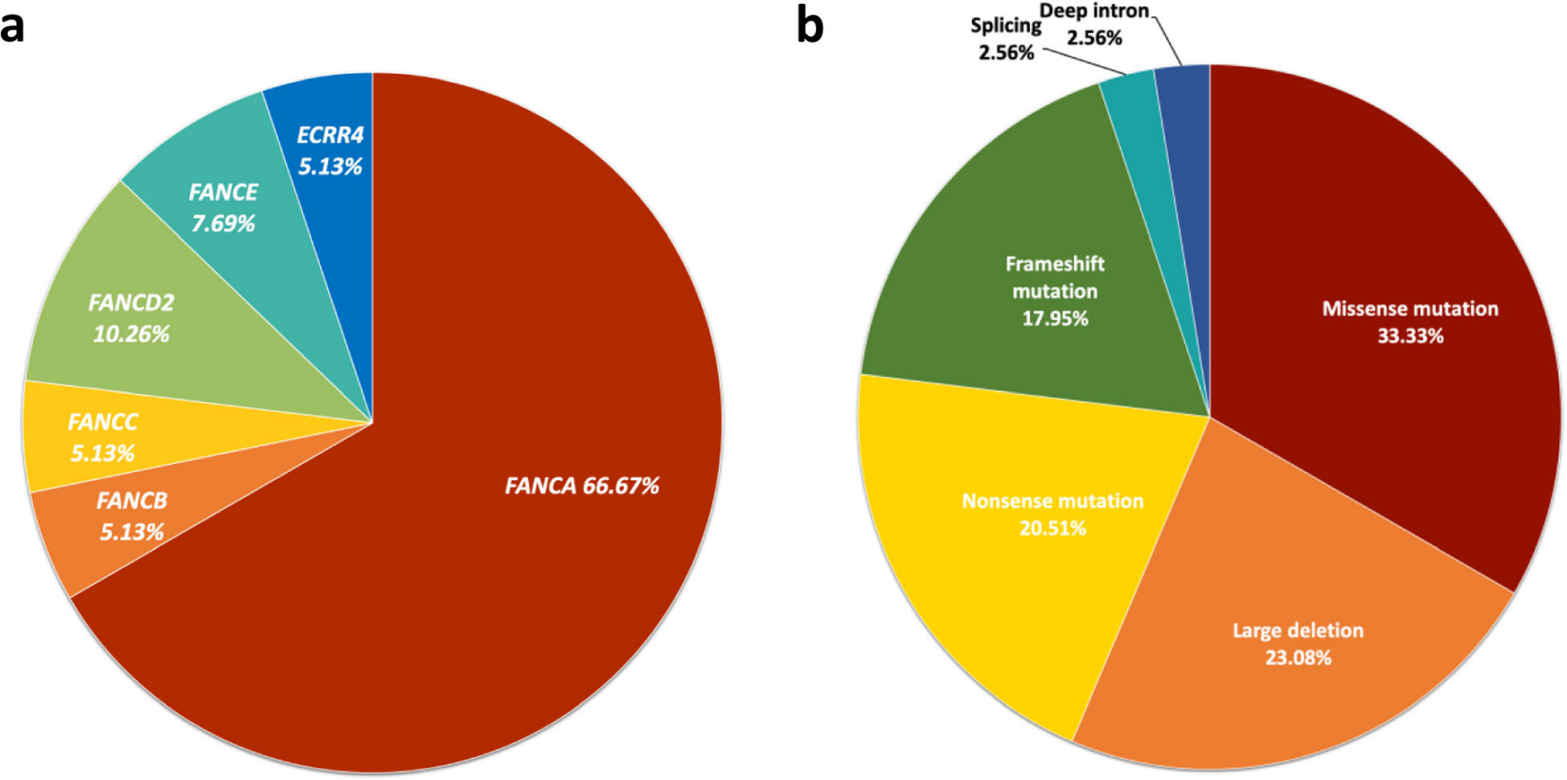
Distribution and composition of the 39 mutations. a. mutation distribution. b. mutation composition

**Fig. 2:**
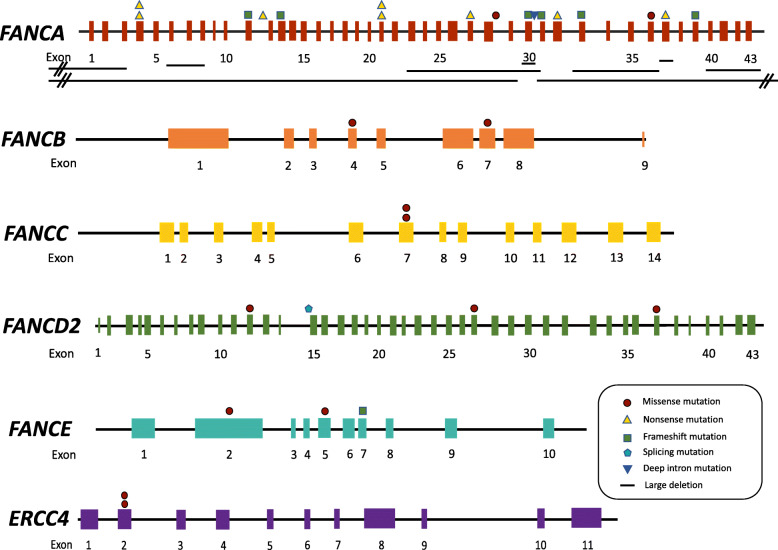
Locations, frequencies, and types of mutations in *FANCA*, *FANCB*, *FANCC*, *FANCD2*, *FANCE*, and *ERCC4* genes. Exons represent by colored rectangles; mutation types are represented by colored patterns; large deletions are represented by the black horizontal bars

A total of 39 mutations were identified involving six different FA genes and composed of 13 missense mutations, nine large deletions, eight nonsense mutations, seven frameshift mutations, one splicing mutations, and one deep intron mutation (Fig. 1, Table 3). All the large deletions were found within the *FANCA* gene. 20 (47.73%) mutations identified in our cohort were novel and the majority of mutations were private except *FANCA* c.367C > T, which was shared by two patients. (Fig. 2, Table 3). We did not find *FANCA* c.2546delC in our cohort, which accounts for over 30% *FANCA* mutations in Japanese and Korean patients [22, 24].

**Table 3:** Mutation details

Case No.	Gene	Mutation 1 (maternal)			Mutation 2 (paternal)		
		Genomic location	cDNA/Protein	Ref./Com.	Genomic location	cDNA/Protein	Ref./Com.
1	*FANCA*	chr16:89833593	c.2557C > T/p.R853X	[20]	chr16:89877396	c.367C > T/p.Q123X	NA
2	*FANCA*	chr16:89815145–89,815,146	c.3270_3271delCT/p.C1090RfsX25	Novel	chr16:89868906–89,875,410	c.792 + 761_c.523-635del	Novel
3	*FANCA*	chr16:89877396	c.367C > T/p.Q123X	NA	chr16:89818822	c.2982-192A > G	[21]
4^a^	*FANCA*	chr16:89842183	c.1867C > T/p.Q623X	Novel	chr16:89842183	c.1867C > T/p.Q623X	Novel
5	*FANCA*	chr16:89804935–89,806,139	c.3935-178_4368 + 74del	Novel	chr16:89819567–89,839,134	c.c.2014 + 545_2982-937del	Novel
6	*FANCA*	chr16:89811185–89,815,741	c.3239 + 397_3626 + 202del	Novel	chr16:89858887	c.1074_1075delGT/p.Y359PfsX49	NA
7	*FANCA*	chr16:89826812–89,919,023	*FANCA* c.2852 + 1545_*SPIRE2* c.646-1671del	Novel	chr16:89825071	c.2894_2895delCT/p.P965RfsX9	Novel
8	*FANCA*	chr16:89780001–89,822,000	*VPS9D1* c.432-877_*FANCA* c.2981 + 2985del	[16]	chr16: 89808940–89,809,954	c.3627-607_3765 + 268del	[16]
9	*FANCA*	chr16:89823177–89,825,446	c.2853-333_2981 + 1808del	Novel	chr16:89809270	c.3703C > T/p.Q1235X	Novel
10	*FANCA*	chr16:89818619	c.2990_2993delGTTA/p.S997MfsX28	NA	chr16:89862229	c.987_990delTCAC/p.H330AfsX4	[22, 23]
11	*FANCA*	chr16:89816286	c.3091C > T/p.Q1031X	NA	chr16:89792569–89,821,767	ZNF276 c.1007-1118_FANCA c.2982-3137del	Novel
12	*FANCA*	chr16:89806417	c.3918dupT/p.Q1307SfsX6	[24]	chr16:89831438	c.2638C > G/p.R880G	NA
13	*FANCA*	chr16:89858941	c.1021C > T/p.Q341X	Novel	chr16:89811412	c.3581C > T/p.P1194L	[24]
14	*FANCB*	chrX:14868651	c.1472 T > A/p.V491E	Novel	–	–	–
15	*FANCB*	chrX:14877390	c.1018C > A/p.Q340K	Novel	–	–	–
16^a^	*FANCC*	chr9:97912346	c.545C > A/p.S182Y	Novel	chr9:97912346	c.545C > A/p.S182Y	Novel
17	*FANCD2*	chr3:10084828	c.983G > A/p.R328Q	NA	chr3:10114634	c.2574 T > G/p.I858M	Novel
18	*FANCD2*	chr3:10132005	c.3713 T > A/p.M1238K	NA	chr3:10089599	c.1279-2A > T	Novel
19	*FANCE*	–	–	NA	chr6:35426215	c.1111C > T/p.R371W	[21, 22, 25]
20	*FANCE*	chr6:35423547	c.272C > T/p.P91L	Novel	chr6:35427467–35,427,470	c.1246_1249delCAAA/p.T417SfsX7	NA
21^a^	*ERCC4*	chr16:14015937	c.257G > A/p.R86H	NA	chr16:14015937	c.257G > A/p.R86H	NA

*NA* not available

^a^ Case 4, Case 16, and Case 21 carry homozygous variants

Among the 21 patients,15 patients carried compound heterozygous mutations, three patients carried homozygous mutations, two patients harbored hemizygous *FANCB* mutations, and one patient with a heterozygous *FANCE* mutation were identified. Biallelic *FANCA* mutations caused 61.90% (13/21) of the cases, followed by monoallelic *FANCB* mutations and FANCD2 mutations, which both constituted 9.52% (2/21) of the cases; and *FANCC*, *FANCE*, and *ERCC4* mutations caused one case each (Table 3). We did not find any case attributed to *FANCG* mutations, which is the second most prevalent responsible gene in East Asian according to Japanese and Korean studies [22, 24]. Despite the limited size of this cohort, we identified two *FANCB* mutations, making it rank one of the most common causative genes in line with the Japanese study [22]. There were three homozygous mutations, *FANCA* c.1867C > T, *FANCC* c.545C > A, and *ERCC4* c.257G > A; the latter two mutations were carried by patients both came from consanguineous families, and the *FANCC* c.545C > A was carried by the only Uyghur patient in our cohort.

Rigorous criteria were adopted in the process of criminal variant identification (Table 3, Table S3). Majority of the patients were assigned with compelling mutations with two exceptions. All mutations were classified as pathogenic or likely pathogenic according to the guideline of ACMG. Case 19 carried compound heterozygous *FANCE* c.1111C > T mutation and *FANCE* c.1317-237C > G mutation. The c.1111C > T mutation was considered pathogenic, but the c.1317-237C > G mutation is an intron variant and classified as uncertain significance, therefore it was excluded in statistics.

### ADLH2 rs671 genotype

12/21 (58.33%) patients in our cohort carried *ALDH2*-G/A genotype, and the other patients were all *ALDH2*-G/G genotype. There was no *ALDH2*-A/A genotype identified (Table 2). The age of BMF onset of *ALDH2*-G/A patients was significantly younger than that of the *ALDH2*-G/G patients (*p* = 0.025, t-test).

### Treatment and outcome

Within the 21 patients, continuous medical records of 19 patients can be retrieved except Case 8 and Case 21, who only came to us once and were excluded in this section. All the 21 patients were eligible for HSCT for they were all transfusion-dependent, and HSCT was performed on 15 patients (71.43%). The numbers of patients accepted HSCT from HLA-matched unrelated donors (MUD), HLA-unmatched unrelated donors (UUD), HLA-haploidentical related (sibling or parental) donors (HRD), and HLA-matched related donors (MRD) were three, four, six, and one, respectively. Another patient accepted HLA-unmatched unrelated cord blood (UUC) HSCT. The other four patients who did not undergo HSCT accepted androgen, cytokine, and/or intermittent transfusion support. All the patients with abnormal karyotype underwent HSCTs. In the HSCT subgroup, 9/15 (60%) were *ALDH2*-G/A genotype. The median follow-up duration was 29 months ranged from 1 month to 68 months. By the end of the study, eight patients (38.10%) have been dead. Seven of them were HSCT-related, mainly severe acute graft-versus-host disease (aGVHD) and/or infections, accounting for 46.67% of the subgroup. One patient who did not receive HSCT died from severe infection (Table 4).

**Table 4:** Treatment and outcomes

Case No.	Therapeutics	Donor & HLA matching	Pre-HSCT conditioning regimen	Outcomes
1	HSCT	UUD; 8/10	Bu + CTX + Flu+Alemtuzumab	Dead (aGVHD, infections)
2	HSCT	UUD; 9/10	Bu + CTX + Flu+ATG + Me-CCNU	Alive
3	HSCT	MUD; 10/10	Bu + Flu+CTX + ATG	Alive
4	HSCT	HRD; 8/10	Bu + CTX + Flu+ATG + Me-CCNU	Alive
5	HSCT	HRD; 6/10		Dead (aGVHD, drug-induced encephalopathy)
			Decitabine+Ara-C + Bu + Flu+ATG + Me-CCNU	
6	Androgen and transfusion	–	–	Alive
7	HSCT	HRD; 7/10		Dead (aGVHD, MODS)
			Decitabine+Ara-C + Bu + Flu+ATG + Me-CCNU	
8	Lost follow-up	–	–	–
9	HSCT	MRD; 10/12	Bu + Flu+CTX + ATG	Dead (aGVHD, septic shock)
10	HSCT	HRD; 5/10		Dead (aGVHD, septic shock)
			Decitabine+Ara-C + Bu + Flu+ATG + Me-CCNU	
11	HSCT	UUC; 5/8	Bu + Flu+CTX + ATG	Dead (aGVHD, pulmonary infection, CMV infection)
12	Androgen, cytokine, transfusion	–	–	Alive
13	Androgen and cytokine	–	–	Alive
14	HSCT	HRD; 7/10	Bu + Flu+CTX + ATG	Dead (aGVHD, TMA, pulmonary infection)
15	HSCT	HRD; 7/10	Bu + Flu+CTX + ATG	Alive
16	Androgen and transfusion	–	–	Dead (pulmonary infection, septic shock)
17	HSCT	MUD; 10/10	Bu + Flu+CTX + ATG	Alive
18	HSCT	UUD; 8/10	Bu + Flu+CTX + ATG	Alive
19	HSCT	UUD; 9/10	TBI + CTX + Flu+ATG	Alive
20	HSCT	MUD; 10/10	Bu + Flu+CTX + ATG	Alive
21	Loss to follow-up	–	–	–

*HSCT* hematologic stem cell transplantation, *UUD* HLA-unmatched unrelated donor, *MUD* HLA-matched unrelated donor, *HRD* HLA-haploidentical related donor, *MRD* HLA-matched related donors, *UUC* HLA-unmatched unrelated cord blood, *Bu* Busulfan, *CTX* cyclophosphamide, *Flu* Fludarabine, *ATG* antithymocyte globulin, *Me-CCNU* Semustine, *TBI* total body irradiation, *aGVHD* acute graft-versus-host disease, *CMV* cytomegalovirus

## Discussion

The 21 patients displayed a wide range of clinical phenotype and genetic variation spectrum that all physiological systems were involved (Table 1), and the responsible mutations were detected in six different genes (Fig. 1, Table 3). In keeping with other studies, bone marrow dysplasia and abnormal karyotypes were prevailing (65 and 42.11%, respectively) and highly consistent [14, 26], denoting the risk of hematologic malignant transformation, especially the ones with aberration in chromosome 7, which is the most prevalent cytogenetic abnormality in pediatric MDS and indicates an adverse long-term outcome even after HSCTs in MDS/AML patients [27]. *ALDH2*-G/A and *ALDH*-A/A genotypes are confirmed to be associated with more severe hematologic phenotype and more adverse outcomes of FA in Asian patients [13, 14]. The same tendency was observed in our cohort, despite there was no patient of *ALDH2*-AA genotype.

All patients in our cohort presented with a more severe hematologic manifestation and the proportion of patients who received HSCTs was higher than that of most studies [3–6, 14, 28]. Although BMF is the typical and most prevalent feature, our data may not reflect the actual behavior of FA since all the patients were referred to our institute seeking for HSCTs. Studies suggest the high HSCT-related mortality in FA patients, of which infection and aGVHD were the two leading causes [5, 28]. In our cohort, 46.67% of HSCT patients died from HSCT-related acute complications. Studies also suggest the overall dismal outcome that 10 years cumulative risk of death was over 22% and the overall survival after 30 years of diagnosis dropped to below 40%; besides, the long-term survival of HSCT patients and non-HSCT patients were comparable [5, 26, 28, 29], partly because HSCT in the context of FA is explicitly challenging. Therefore, even with the optimized pre-HSCT conditioning regimens like the reduced intensity and the combination of fludarabine, meticulousness is needed in decision-making. Whether HSCT is the best treatment strategy depends much on the severity of cytopenia and the hematologic adverse events of a given patient and the type of donor he/she could get.

The cumulative incidence of leukemia and solid tumors in the middle age of FA patients was reported to be ~ 20% and ~ 30%, respectively [4–6, 30]. In our cohort, no patient developed hematologic or solid malignancies during the follow-up up to date except the ones initially diagnosed as AML (Case 5) and MDS (Case 10), but the longest follow-up in our cohort was only 5.5 years, which may not be long enough for the malignant phenotype to emerge.

## Conclusions

Although this study is limited by its cohort size, it is still informative and enriches the knowledge on Chinese FA patients which was nearly a barren. Here we thoroughly investigated the clinical manifestations, morphologic and cytogenetic changes, genetic basis, and outcomes of 21 Chinese FA patients. Our data displayed a broad phenotypic and genetic variant spectrum of Chinese FA patients, the disappointing outcomes which need improving, and highlighted the urgency of nationwide multicenter studies to reveal the mask of Chinese FA patients and optimize the clinical management.

## Supplementary information

**Additional file 1.** Supplementary methods.

**Additional file 2: Table S1.** Details of 22 FA-related genes.

**Additional file 3.** Data of hromosome breakage test.

**Additional file 4: Table S3.** Mutation interpretations.

## Data Availability

The datasets generated during the current study are available in the Sequence Read Archive (SRA) repository of the National Center for Biotechnology Information [accession number: PRJNA631475; https://www.ncbi.nlm.nih.gov/bioproject/631475]. The reference datasets used in this study is human reference genome (hg19).

## Abbreviations

ACMG: American College of Medical Genetics and Genomics
AGE: Agarose gel electrophoresis
aGVHD: Acute graft-versus-host disease
ALDHs: Aldehyde dehydrogenases
AML: Acute myeloid leukemia
BM: Bone marrow
BMF: Bone marrow failure
BWA: Burrow-Wheeler Aligner
CNVs: Copy number variants
FA: Fanconi anemia
HLA: Human leukocyte antigen
HRD: HLA-haploidentical related donors
HSCT: Hematopoietic stem cell transplantations
ICLs: Interstrand crosslinks
InDels: Insertions/deletions
MAF: Minimal allele frequency
MDS: Myelodysplastic syndrome
MRD: HLA-matched related donors
MUD: HLA-matched unrelated donors
PB: Peripheral blood
PCR: Polymerase chain reaction
SNVs: Single nucleotide variants
THS: Targeted high-throughput sequencing
UUC: HLA-unmatched unrelated cord blood
UUD: HLA-unmatched unrelated donors
WGS: Whole genomic sequencing
