# Supplementary material for: Comprehensive analysis on phenotype and genetic basis of Chinese Fanconi anemia patients: dismal outcomes call for nationwide studies
Source: BMC Med Genet. 2020 Jun 1;21:118. doi: 10.1186/s12881-020-01057-3 (PMC7268325; doi:10.1186/s12881-020-01057-3)
Supplement: Supplementary file 1 — Additional file 1. Supplementary methods. [file 12881_2020_1057_MOESM1_ESM.docx]

**Comprehensive analysis on phenotype and genetic basis of Chinese Fanconi anemia patients: dismal outcomes call for nationwide studies**

Nie et al.

**Supplementary methods:**

**Chromosome breakage test**

8-10 mL peripheral blood sample from the patient and 20 healthy controls were collected and anticoagulated with sodium heparin. Lymphocyte was obtained using Ficoll-Paque density gradient media and cultured in medium added with phytohaemagglutinin (PHA) and mitomycin C (MMC) for 72 hours. Concentrations of MMC were 0 ng/ml, 50 ng/mL and 100 ng/ml, respectively. Cultures were exposed to Colcemid before harvest. In the harvest process, the cells were transferred into a hypotonic solution and fixed with glacial acetic acid and methanol. Metaphases were stained with Giemsa’s, and 100 metaphases of the proband and 20 metaphases of the controls in each culture with different MMC concentrations were analyzed.
