# Supplementary material for: Comprehensive analysis on phenotype and genetic basis of Chinese Fanconi anemia patients: dismal outcomes call for nationwide studies
Source: BMC Med Genet. 2020 Jun 1;21:118. doi: 10.1186/s12881-020-01057-3 (PMC7268325; doi:10.1186/s12881-020-01057-3)
Supplement: Supplementary file 2 — Additional file 2: Table S1. Details of 22 FA-related genes. [file 12881_2020_1057_MOESM2_ESM.docx]

Table S1 Details of 22 FA-related genes

| Gene | HGNC symbol | #MIM | Cytogenetic location | Genomic coordinate (GRCh37) | mRNA reference | Phenotype | Inheritance |
| --- | --- | --- | --- | --- | --- | --- | --- |
| *FANCA* | *FANCA* | 607139 | 16q24.3 | chr16:89803957-89883065 | NM_000135 | FA | AR |
| *FANCB* | *FANCB* | 300515 | Xp22.2 | chrX:14861529-14891191 | NM_152633 | FA | XLR |
| *FANCC* | *FANCC* | 613899 | 9q22.32 | chr9:97861336-98079991 | NM_000136 | FA | AR |
| *FANCD1* | *BRCA2* | 600185 | 13q13.1 | chr13:32889611-32973805 | NM_000059 | FA, FBOC | AR, AD |
| *FANCD2* | *FANCD2* | 613984 | 3p25.3 | chr3:10068098-10143614 | NM_033084 | FA | AR |
| *FANCE* | *FANCE* | 613976 | 6p21.31 | chr6:35420138-35434880 | NM_021922 | FA | AR |
| *FANCF* | *FANCF* | 613897 | 11p14.3 | chr11:22644079-22647387 | NM_022725 | FA | AR |
| *FANCG* | *FANCG* | 602956 | 9q13.3 | chr9:35073832-35080013 | NM_004629 | FA | AR |
| *FANCI* | *FANCI* | 611360 | 15q26.1 | chr15:89787180-89860492 | NM_001113378 | FA | AR |
| *FANCJ* | *BRIP1* | 605882 | 17q23.2 | chr17:59758627-59940882 | NM_032043 | FA | AR |
| *FANCL* | *FANCL* | 608111 | 2p16.1 | chr2:58386378-58468507 | NM_001114636 | FA | AR |
| *FANCM** | *FANCM* | 609644 | 14q21.2 | chr14:45605143-45670093 | NM_020937 | Cancer predisposition | AR |
| *FANCN* | *PALB2* | 610355 | 16p12.2 | chr16:23614488-23652631 | NM_024675.3 | FA | AR |
| *FANCO* | *RAD51C* | 602774 | 17q22 | chr17:56769934-56811703 | NM_058216 | FA-like | AR |
| *FANCP* | *SLX4* | 613278 | 16p13.3 | chr16:3631182-3661599 | NM_032444 | FA | AR |
| *FANCQ* | *ERCC4* | 133520 | 16p13.12 | chr16:14014014-14046202 | NM_005236 | FA | AR |
| *FANCR* | *RAD51* | 179617 | 15q15.1 | chr15:40986972-41024354 | NM_133487 | FA-like | AD |
| *FANCS* | *BRCA1* | 113705 | 17q21.31 | chr17:41196312-41277500 | NM_007300 | FA-like, FBOC | AR, AD |
| *FANCT* | *UBE2T* | 610538 | 1q32.1 | chr1:202300785-202311108 | NM_014176 | FA | AR |
| *FANCU* | *XRCC2* | 600375 | 7q36.1 | chr7:152341864-152373250 | NM_005431 | FA | AR |
| *FANCV* | *MAD2L2* | 604094 | 1p36.22 | chr1:11734537-11751707 | NM_001127325 | FA | AR |
| *FANCW* | *RFWD3* | 614151 | 16q23.1 | chr16:74655292-74700779 | NM_018124 | FA | AR |

FA, Fanconi anemia; FBOC, familial breast and ovarian cancer; AR, autosomal recessive inheritance; AD, autosomal dominant inheritance; XLR, X-linked recessive inheritance.
