# Supplementary material for: Comprehensive analysis on phenotype and genetic basis of Chinese Fanconi anemia patients: dismal outcomes call for nationwide studies
Source: BMC Med Genet. 2020 Jun 1;21:118. doi: 10.1186/s12881-020-01057-3 (PMC7268325; doi:10.1186/s12881-020-01057-3)
Supplement: Supplementary file 4 — Additional file 4: Table S3. Mutation interpretations. [file 12881_2020_1057_MOESM4_ESM.docx]

Table S3 Mutation interpretations

|  |  | Mutation 1 (maternal) | | | Mutation 2 (paternal) | | |
| --- | --- | --- | --- | --- | --- | --- | --- |
| Case No. | Gene | cDNA/Protein | Evidence | Interpretation | cDNA/Protein | Evidence | Interpretation |
| 1 | *FANCA* | c.2557C>T/p.R853X | PVS1+PM3+PP4+PP5 | Pathogenic | c.367C>T/p.Q123X | PVS1+PM3+PP4 | Pathogenic |
| 2 | *FANCA* | c.3270_3271delCT/p.C1090RfsX25 | PVS1+PM3+PP4 | Pathogenic | c.792+761_c.523-635del | PVS1+PM3+PP4 | Pathogenic |
| 3 | *FANCA* | c.367C>T/p.Q123X | PVS1+PM3+PP4 | Pathogenic | c.2982-192A>G | PM2+PM3+PP3+PP4+PP5 | Likely pathogenic |
| 4* | *FANCA* | c.1867C>T/p.Q623X | PVS1+PM3+PP4 | Pathogenic | c.1867C>T/p.Q623X | PVS1+PM3+PP4 | Pathogenic |
| 5 | *FANCA* | c.3935-178_4368+74del | PVS1+PM3+PP4 | Pathogenic | c.c.2014+545_2982-937del | PVS1+PM3+PP4 | Pathogenic |
| 6 | *FANCA* | c.3239+397_3626+202del | PVS1+PM3+PP4 | Pathogenic | c.1074_1075delGT/p.Y359PfsX49 | PVS1+PM3+PP4 | Pathogenic |
| 7 | *FANCA* | *FANCA* c.2852+1545_*SPIRE2* c.646-1671del | PVS1+PM3+PP4 | Pathogenic | c.2894_2895delCT/p.P965RfsX9 | PVS1+PM3+PP4 | Pathogenic |
| 8 | *FANCA* | *VPS9D1* c.432-877_*FANCA* c.2981+2985del | PVS1+PS3+PM3+PP4 | Pathogenic | c.3627-607_3765+268del | PVS1+PS3+PM3+PP4 | Pathogenic |
| 9 | *FANCA* | c.2853-333_2981+1808del | PVS1+PM3+PP4 | Pathogenic | c.3703C>T/p.Q1235X | PVS1+PM3+PP4 | Pathogenic |
| 10 | *FANCA* | c.2990_2993delGTTA/p.S997MfsX28 | PVS1+PM3+PP4 | Pathogenic | c.987_990delTCAC/p.H330AfsX4 | PVS1+PM3+PP4 | Pathogenic |
| 11 | *FANCA* | c.3091C>T/p.Q1031X | PVS1+PM3+PP4 | Pathogenic | ZNF276 c.1007-1118_FANCA c.2982-3137del | PVS1+PM3+PP4 | Pathogenic |
| 12 | *FANCA* | c.3918dupT/p.Q1307SfsX6 | PVS1+PM3+PP4+PP5 | Pathogenic | c.2638C>G/p.R880G | PM2+PM3+PP3+PP4 | Likely pathogenic |
| 13 | *FANCA* | c.1021C>T/p.Q341X | PVS1+PM3+PP4 | Pathogenic | c.3581C>T/p.P1194L | PM2+PM1+PM3+PP3+PP4+PP5 | Likely pathogenic |
| 14 | *FANCB* | c.1472T>A/p.V491E | PM2+PM3+PP3+PP4 | Likely pathogenic | — | — | — |
| 15 | *FANCB* | c.1018C>A/p.Q340K | PM2+PM3+PP3+PP4 | Likely pathogenic | — | — | — |
| 16* | *FANCC* | c.545C>A/p.S182Y | PM2+PM1+PM3+PP3+PP4 | Likely pathogenic | c.545C>A/p.S182Y | PM2+PM1+PM3+PP3+PP4 | Likely pathogenic |
| 17 | *FANCD2* | c.983G>A/p.R328Q | PM2+PM3+PP3+PP4 | Likely pathogenic | c.2574T>G/p.I858M | PM2+PM3+PP3+PP4 | Likely pathogenic |
| 18 | *FANCD2* | c.3713T>A/p.M1238K | PM2+PM1+PM3+PP3+PP4 | Likely pathogenic | c.1279-2A>T | PVS1+PM3+PP4 | Pathogenic |
| 19 | *FANCE* | — | — | — | c.1111C>T/p.R371W | PM2+PM1+PM3+PP3+PP4+PP5 | Likely pathogenic |
| 20 | *FANCE* | c.272C>T/p.P91L | PM2+PM3+PP3+PP4 | Likely pathogenic | c.1246_1249delCAAA/p.T417SfsX7 | PVS1+PM3+PP4 | Pathogenic |
| 21* | *ERCC4* | c.257G>A/p.R86H | PM2+PM1+PM3+PP3+PP4 | Likely pathogenic | c.257G>A/p.R86H | PM2+PM1+PM3+PP3+PP4 | Likely pathogenic |

* Case 4, Case 16, and Case 21 carry homozygous variants.
